# A detective of intramural ectopic pregnancy: The use of pituitrin under hysteroscopy combined with laparoscopy

**DOI:** 10.1097/MD.0000000000033379

**Published:** 2023-03-24

**Authors:** Yanchao Guo, Tongfu Feng, Xin Du

**Affiliations:** a Department of Obstetrics and Gynecology, Medical College of Wuhan University of Science and Technology, Wuhan, Hubei Province, China; b Department of Gynecology, Maternal and Child Health Hospital of Hubei Province, Wuhan, Hubei Province, China.

**Keywords:** hysteroscopy, intramural ectopic pregnancy, laparoscopy, pituitrin

## Abstract

Intramural pregnancy is a rare ectopic pregnancy in which the gestational sac is implanted between the muscle walls. Due to the lack of specific clinical manifestations, it is easy to misdiagnose or miss them. If it is allowed to get worse, the uterus will burst, and there will be a lot of bleeding in the later stages, which could lead to the death of the patient.

**Patient concerns:**

The patient had no history of uterine surgery, embryo transplantation, or any other operations. She complained of having abdominal distention and swelling of the waist but no vaginal bleeding or lower abdomen discomfort.

**Diagnoses:**

According to her transvaginal ultrasonography, we highly suspected ectopic pregnancy. Hysteroscopy combined with laparoscopy is an effective treatment option that can prevent life-threatening problems. During the surgery, pituitrin helped find the gestational sac, and the pathology report confirmed that it was an intramural pregnancy.

**Interventions:**

Hysteroscopy combined with laparoscopy is an effective treatment option that can prevent life-threatening problems. During the surgery, we used pituitrin to help find the gestational sac. The use of pituitrin can minimize bleeding during a uterine operation and indicate the location of an intramural pregnancy, helping surgeons to complete the operation successfully.

**Outcomes:**

The patient recovered quickly and was discharged on the 4th day after surgery, with a significant decrease in human chorionic gonadotrophin (HCG) levels from 14,792.26 mIU/mL before surgery to 1071.40 mIU/mL at discharge. During the follow up, her HCG level dropped to 50.90 mIU/mL on the 14th day after the surgery. She monitored the HCG levels intermittently until they fell within the normal range.

**Lessons:**

Intramural pregnancy is a rare form of ectopic pregnancy, and it is difficult to diagnose early on. This may result in uterine rupture or even life-threatening hemorrhage. If an intramural pregnancy is suspected in early pregnancy, hysteroscopy combined with laparoscopy is advised, and if necessary, low-dose posterior pituitary hormone can enhance uterine contractions and better reveal the position of the gestational sac within the uterine wall.

## 1. Introduction

Intramural pregnancy is a rare ectopic pregnancy in which the gestational sac is implanted between the muscle walls. Its incidence is <1% of the total number of ectopic pregnancies.^[1]^ It was first reported by Doederlein et al in 1913.^[2]^ The cause of intramural pregnancy is unclear, and the possible risk factors include prior uterine trauma, adenomyosis, pelvic surgery, and in vitro fertilization. Uterine trauma results in a sinus tract within the endometrium and may be the consequence of a cesarean section, myomectomy, dilation, or curettage.^[3]^ There have been 2 cases reported in the literature of gestation (G) continuing without rupture until more than 30 weeks G, with neonatal survival.^[4,5]^ Due to the lack of specific clinical manifestations, it is easy to misdiagnose or miss them. If it is allowed to develop, uterine rupture and massive bleeding will occur in the later stages, endangering the lives of patients. In this case report, we describe a case of intramural pregnancy without a history of uterine surgery, embryo transplantation, or any other operations. During the surgery, we used pituitrin to help find the gestational sac. The medical ethics committee of Hubei Maternal and Child Health Care Hospital’s approval was obtained.

## 2. Case presentation

**Figure 1.**
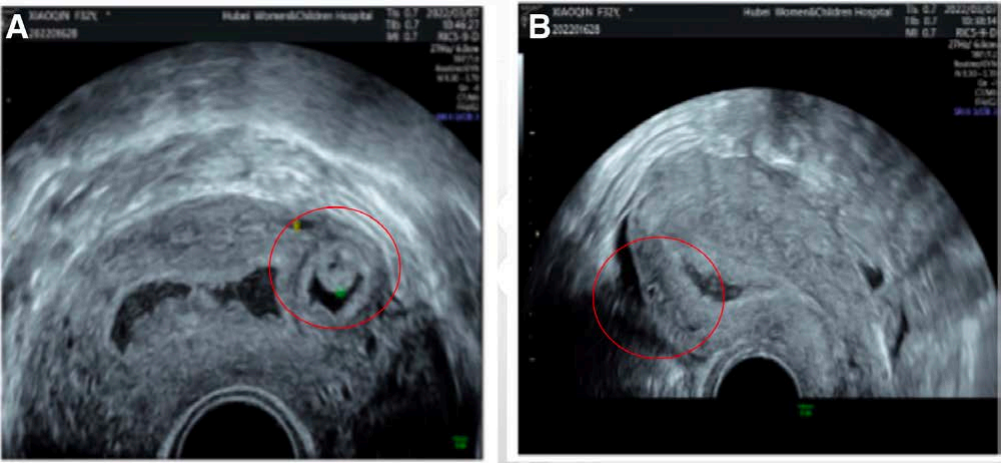
Transvaginal ultrasonography showed a gestational sac inside in the myometrium below the right uterine horn with blood flow signals around, and there was a small passage connecting the intramural gestational sac with the uterine cavity.

**Figure 2.**
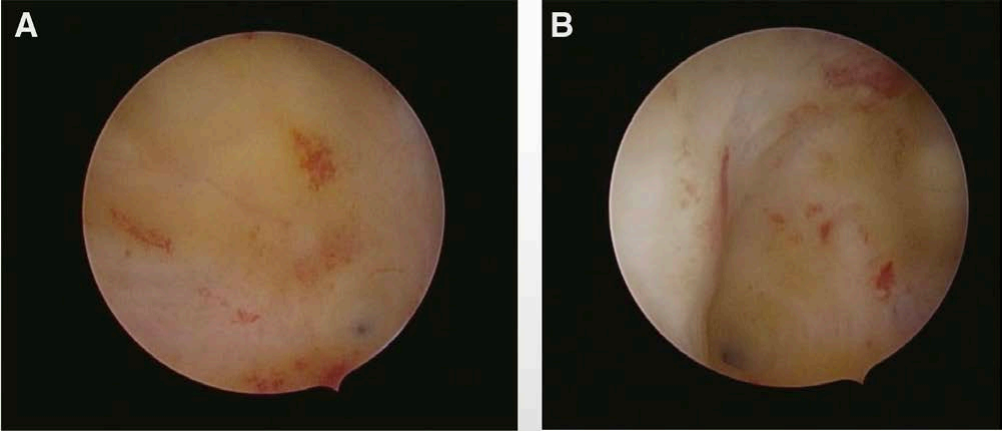
Intraoperative hysteroscopy showed that the size and shape of the uterine cavity were normal, and both ostia from fallopian tubes were seen. There were no small holes or defects in it.

**Figure 3.**
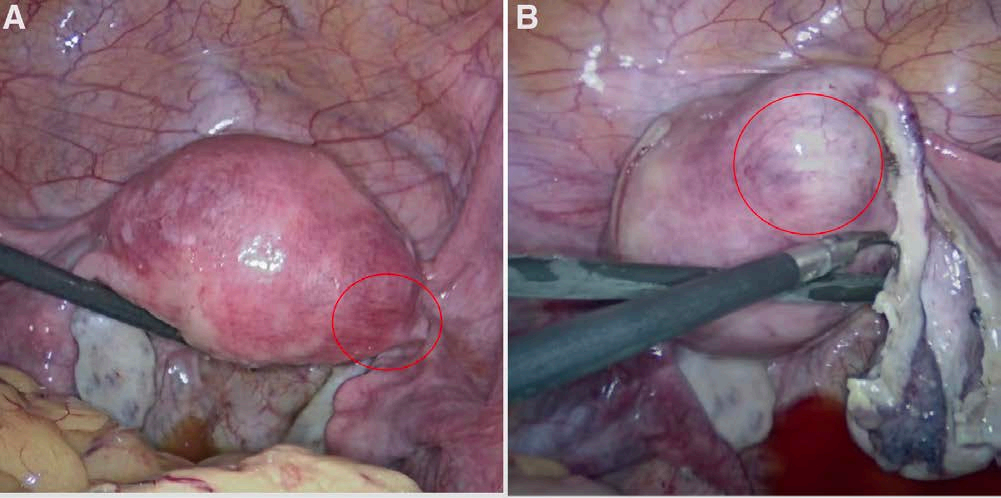
(A) Under laparoscopy, the size of the uterus was normal, and the right uterine horn was inapparently swollen. (B) After injection of pituitrin, the color of the uterus became lilac and the posterior wall of the uterine fundus was swollen near the right uterine corner. The lesion is indicated by the red circle.

**Figure 4.**
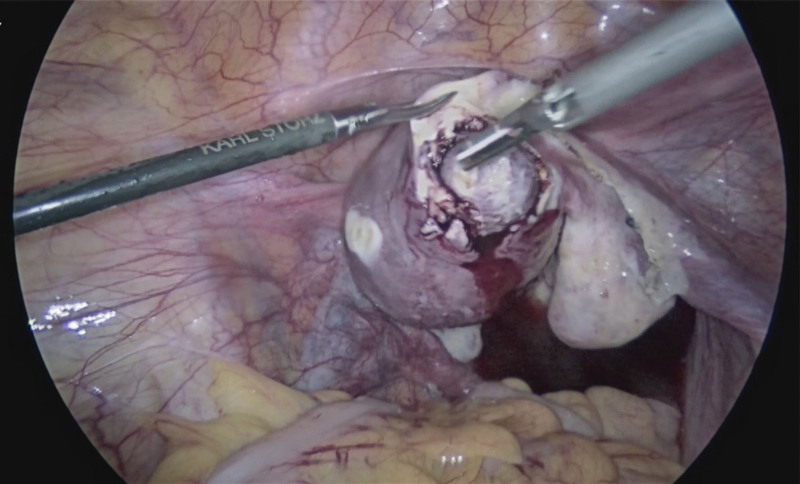
Laparoscopic finding showed an intramural pregnancy in the right uterine horn.

**Figure 5.**
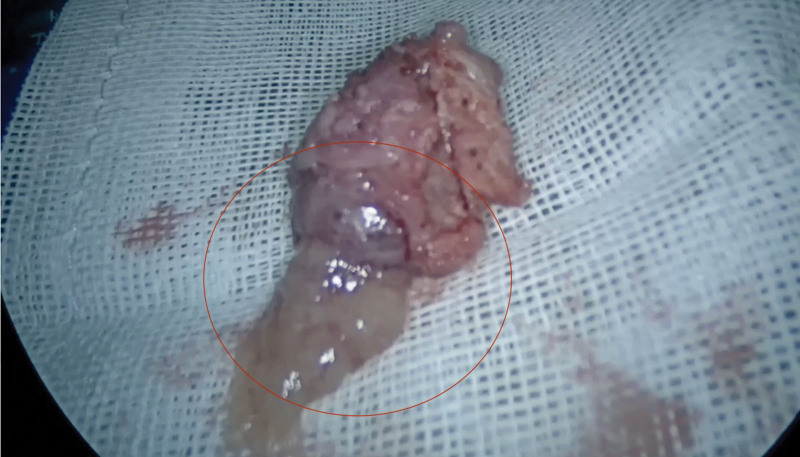
The visible chorionic villi were found.

**Figure 6.**
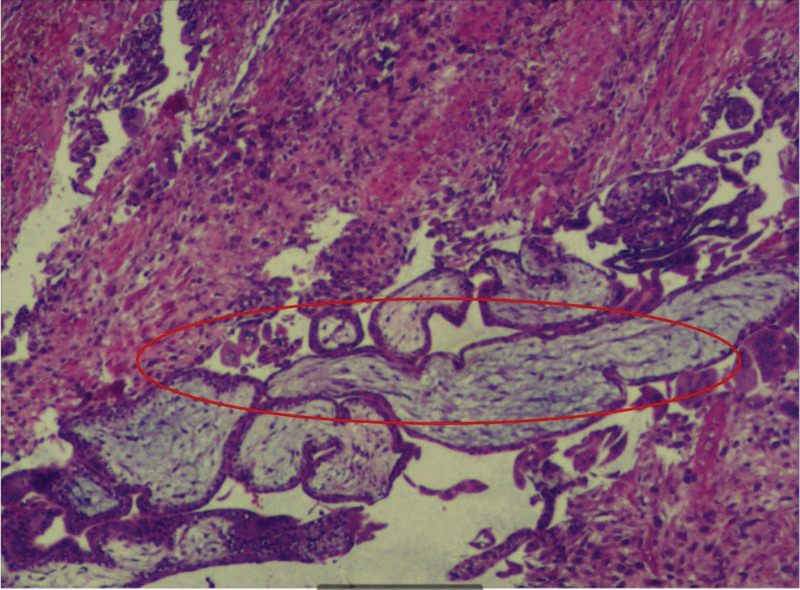
The pathological report confirmed the presence of chorionic villi in the uterine myometrium. The red circle points to the chorionic villi.

We presented the case of a 32-year-old woman, G3P2A0, with 2 spontaneous births and regular menstrual periods. She had a history of dysmenorrhea, and this pregnancy was natural. She had never had uterine surgery, an embryo transplant, or any other type of surgery. The patient’s last menstrual period was January 28, 2022, and the urine pregnancy test was positive on March 4, 2022. She complained of abdominal distension and waist swelling, but no vaginal bleeding or lower abdomen discomfort. On March 5, 2022, she went to our hospital for treatment, and we checked the pelvic ultrasound. Her transvaginal ultrasonography showed a 1.0 * 0.8 * 0.7 cm yolk sac inside the myometrium below the right uterine horn with blood flow signals around it, and there was a small passage connecting the intramural gestational sac with the uterine cavity (Fig. 1). However, her physical examination showed no obvious positive signs. After culdocentesis, 1 mL of pale red liquid was extracted. We had a strong suspicion of ectopic pregnancy, particularly in cases of right tubal interstitial or right uterine horn intramural pregnancy. After all, there was a chance of B-ultrasound errors, so the possibility of a right interstitial tubal pregnancy cannot be completely ruled out. Therefore, the patient requested that the right fallopian tube be removed at the same time during the surgery. Considering the risks of intramural pregnancy, we decided to proceed with exploratory laparoscopy and hysteroscopy. Under hysteroscopy, no sign of the gestational sac was found. The size and shape of the uterine cavity were normal, and both ostia from the fallopian tubes were seen (Fig. 2). There were no small holes or defects in it. Under laparoscopy, the size of the uterus was normal, and the right uterine horn was barely swollen (Fig. 3A). There was no active bleeding in the pelvic cavity. Both fallopian tubes and ovaries looked very normal. After a routine salpingectomy, the uterine body was injected with diluted pituitrin 3U. After several seconds, it was found that the color of the uterus had changed to lilac and the posterior wall of the uterine fundus was swollen by about 3.5 * 3.0 * 3.0 cm near the right uterine corner, which was cystic (Fig. 3B). The serosa of the uterine horn was electric coagulated by biclamp and cut with an ultrasonic knife, and then the pregnancy sac jumped out (Fig. 4). The pregnancy sac was separated and stripped with an ultrasonic knife. The specimen was taken out and rinsed, and the visible chorionic villi were found (Fig. 5). The diagnosis of intrauterine pregnancy was confirmed intraoperatively. Then we excised the lesion and carefully sutured the repair. The uterus contracted nicely throughout the procedure due to the administration of pituitrin, and the estimated blood loss was around 10 mL. The existence of chorionic villi in the myometrium was verified by the pathology report (Fig. 6), validating the diagnosis of an intramural pregnancy but not finding evidence of adenomyosis. The patient recovered quickly and was discharged on the 4th day after surgery, with a significant decrease in human chorionic gonadotrophin (HCG) levels from 14,792.26 mIU/mL before surgery to 1071.40 mIU/mL at discharge. During the follow-up, her HCG level dropped to 50.90 mIU/mL on the 14th day after the surgery. She monitored the HCG levels intermittently until they fell within the normal range.

## 3. Discussion and conclusion

Intramural pregnancy is a rare type of ectopic pregnancy, and its exact cause is unknown. At this time, the following factors are considered to be mainly responsible for intramural pregnancy: prior uterine trauma (such as cesarean section, myomectomy, hysteroscopy, uterine curettage, and a history of uterine perforation), adenomyosis, pelvic surgery, and in vitro fertilization.^[6]^

In this case, the patient delivered vaginally twice, suffered from dysmenorrhea, and denied having had any previous intrauterine operations, surgery, adenomyosis, or in vitro fertilization. Therefore, we hypothesized that her intramural pregnancy and dysmenorrhea were related. As we know, dysmenorrhea is divided into 2 categories: primary and secondary. Primary dysmenorrhea has no underlying organic disease, whereas secondary dysmenorrhea generally has an underlying organic disease such as endometriosis, adenomyosis, or uterine myoma.^[7]^ If the dysmenorrhea is secondary, the intraoperative and pathology reports will reveal relevant signs and evidence. However, intraoperative exploration revealed no ovarian or peritoneal endometriosis, and the uterus did not have a large size like adenomyosis or adenomyoma. In addition, the pathologic diagnosis did not reveal adenomyosis. The cause of primary dysmenorrhea is unclear.^[8]^ The present study did not find an association between primary dysmenorrhea and intramural pregnancy. Obviously, our patient had no remarkable history of gynecological diseases.^[9]^ Even though her B-ultrasound showed a tiny sinus tract connecting the gestational sac to the uterine cavity, which helped the embryo get into the myometrium,^[9]^ careful hysteroscopy did not find any visible sinus tracts during the procedure. It is likely that the formation of microscopic fistulae is an important factor in the pathogenesis of intramural pregnancy.^[10]^ As a result, we concluded that there was a link between the development of this intramural pregnancy and the construction of a sinus tract that is not apparent to the human eye, though the cause of this sinus tract formation is unknown. Given the rarity of intramural pregnancies, it is crucial that additional instances be investigated etiologically and that data be gathered in order to establish the connection between sinus formation and intramural pregnancy. We speculate that the occurrence of intramural pregnancy may also be attributable to the following factors: Similar to an invasive hydatidiform mole, the cytotrophoblast possessed a high erosive capacity. As a result, it invaded the muscular layer. Congenital defects of the intima or hypoplasia of the basal decidua, which allow the blastocyst to penetrate the muscularis following positioning, adhesion, and implantation into the uterine cavity. Intrauterine infection and multiparity have the potential to damage the intima.

Intramural pregnancy often presents with nonspecific clinical symptoms, including mild vaginal bleeding and abdominal pain; however, some patients may be asymptomatic.^[11]^ It is difficult to make an early diagnosis of an intramural ectopic pregnancy. This could lead to uterine rupture or even life-threatening bleeding; in most cases, surgical intervention and even hysterectomy are required.^[12]^ Diagnostic modalities may include sonography, computed tomography scanning, and magnetic resonance imaging.^[3]^ the diagnosis was sometimes made based on postoperative pathology results; some cases were not found until the rupture of the conception.^[13]^ Sonography is the most common tool for diagnosing the disease. Wang Junmei et al^[14]^ indicated that with the development of sonographic imaging, particularly 3-dimensional imaging, it may become a valuable diagnostic tool for the early identification of this disease.

In our case, B-ultrasound suspected intramural pregnancy at 5 weeks G, and laparoscopy combined with hysteroscopy made a definite diagnosis and treated it. During the procedure, pituitrin was used. Pituitrin was used to track and locate the position of the gestational sac, induce uterine contractions, lessen uterine blood loss during surgery. According to a study, intramural pregnancy can be successfully managed using laparoscopy or hysteroscopy in the early stages of pregnancy with a blood loss of <500 mL in all cases without blood transfusion.^[10]^

Hysteroscopy combined with laparoscopy can check for the possibility of intrauterine pregnancy during surgery, and diagnosis and treatment can be performed concurrently to reduce intraoperative bleeding. Hysteroscopy combined with laparoscopy also can improve the survival rate of patients, create favorable conditions for the diagnosis and treatment of intramural pregnancy, enhance the precision of diagnosis, the efficacy of treatment, and the safety of surgery. It deserves clinical promotion and use. Therefore, we recommend that, with the patient’s informed consent, hysteroscopy combined with laparoscopy is a realistic and efficient treatment option when a B-ultrasound strongly suggests an intrauterine ectopic pregnancy. We also suggest using pituitrin. The use of low-dose pituitrin can enhance uterine contraction, reduce bleeding during uterine surgery, and reveal the position of the gestational sac within the uterine wall, allowing surgeons to perform the procedure more effectively. For patients, this method can also reduce the cost and duration of hospitalization, as well as the time required for recovery after surgery.

## Author contributions

**Supervision:** Tongfu Feng.

**Writing – original draft:** Yanchao Guo.

**Writing – review & editing:** Tongfu Feng, Xin Du.
